# Supplementary material for: Composites of Nucleic Acids and Boron Clusters (C2B10H12) as Functional Nanoparticles for Downregulation of EGFR Oncogene in Cancer Cells
Source: Int J Mol Sci. 2021 May 4;22(9):4863. doi: 10.3390/ijms22094863 (PMC8125477; doi:10.3390/ijms22094863)

Composites of nucleic acids and boron clusters ($C_2B_{10}H_{12}$) as functional nanoparticles for downregulation of EGFR oncogene in cancer cells

Damian Kaniowski [1], Katarzyna Ebenryter-Olbińska [1], Katarzyna Kulik [1], Justyna Suwara [1], Wojciech Cypryk [1], Agata Jakóbik-Kolon [2], Zbigniew Leśnikowski [3] and Barbara Nawrot [1,*]

[1] Centre of Molecular and Macromolecular Studies, Polish Academy of Sciences, Sienkiewicza 112, 90-363 Lodz, Poland; dkanio@cbmm.lodz.pl (D.K.); kebenryt@cbmm.lodz.pl (K.E.O.); kpieta@cbmm.lodz.pl (K.K.); jmilczar@cbmm.lodz.pl (J.S.); wcypryk@cbmm.lodz.pl (W.C.)
[2] Department of Inorganic, Analytical Chemistry and Electrochemistry, Faculty of Chemistry, Silesian University of Technology, Krzywoustego 6, 44-100 Gliwice, Poland; agata.jakobik-kolon@polsl.pl (A.J.K.)
[3] Institute of Medical Biology, Polish Academy of Sciences, Laboratory of Medicinal Chemistry, Lodowa 106, 92-232 Lodz, Poland; zlesnikowski@cbm.pan.pl (Z.L.)
* Correspondence: bnawrot@cbmm.lodz.pl; Tel.: +48-42-6803248

Supplementary Materials:

Table S1. Sequence and spectral (MS, UV) and chromatographic data of tripeds 1,2,FL-1, FL-2, ASO-22 and RNA.

Comp. No.	Oligonucleotide sequence	$MW_{calc.}$	*m/z*	Rt [min][a]	λ_{max} [nm]
**ASO-22**	5’-d(TTT CTT TTC CTC CAG AGC CCGA)-3’	6612.28	6612.07[b]	12.94	263
**RNA**	3’-AAA GAA AAG GAG GUC UCG GGCU-5’	7167.38	7168.27	15.3	256
**ASO-C**	5’-d(ATG AAG GTT CAA TCT GAT TTT)- 3’	6450.3	6450.12	12.76	259
**1**	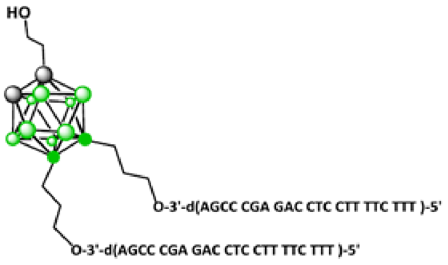	13652.92	13653.55[c]	13.38	261

**2**	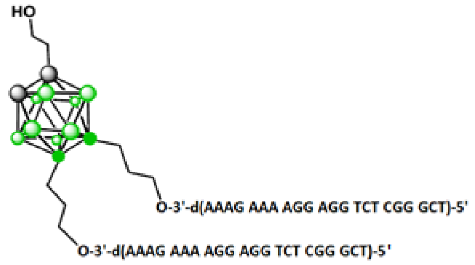	14143.29	14143.75[c]	12.47	258
**FL-1**	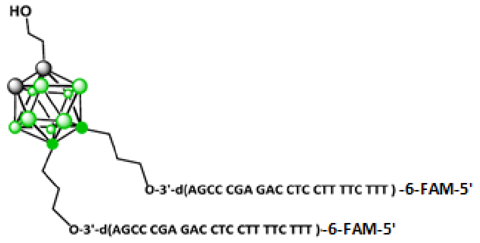	14724.91	14718.02[c]	14.30	261 / 494
**FL-2**	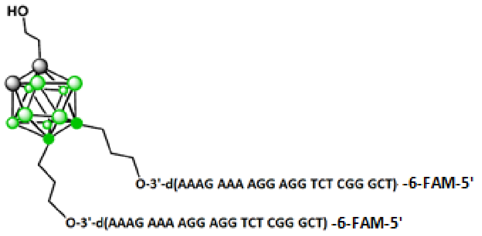	15215.30	15208.50[c]	13.69	257 / 494

[a] The RP-HPLC conditions are described in the Materials and methods section. [b] The m/z ratio of an ion is measured by MALDI-TOF MS. [c] The m/z ratio of an ion is measured by ESI-Q-TOF MS.

Figure S1. RP-HPLC preparative (A) and analytical (B) analysis of triped FL-1

A

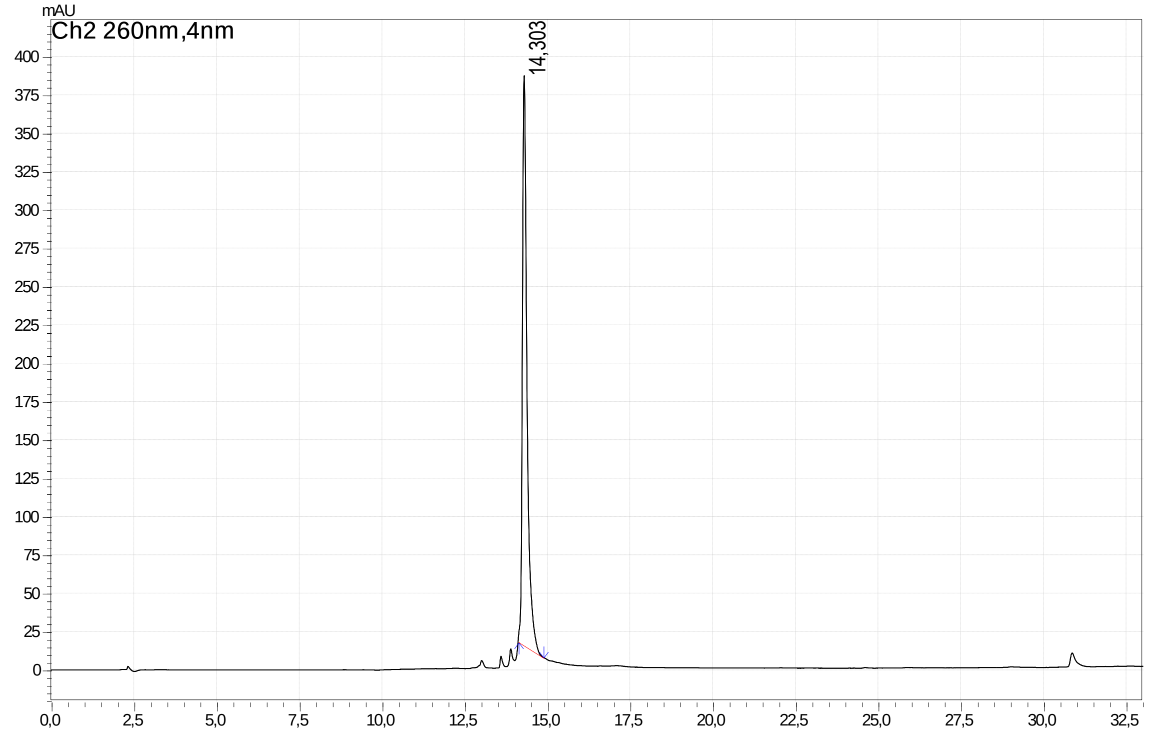

B

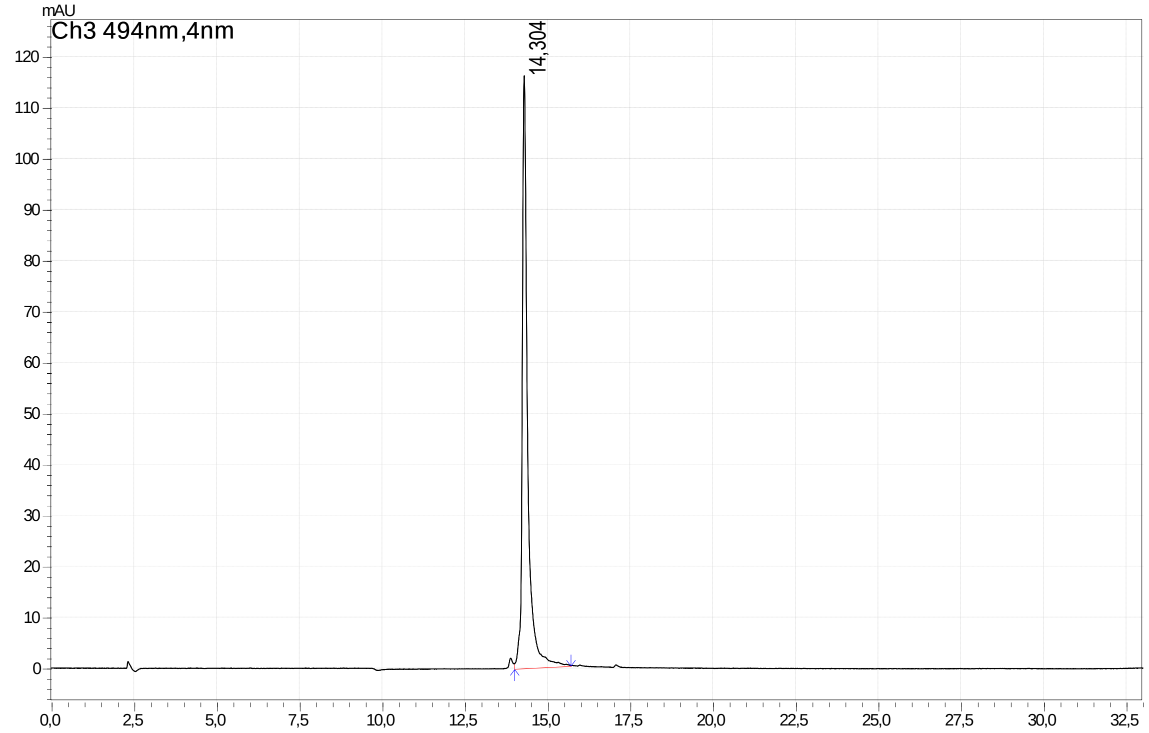

RP-HPLC conditions were as follow: the buffer A (0.1 M CH_3COONH_4) and buffer B (100% CH_3CN). The buffer B gradient: 0→2 min 0%; 2→25 min 0-45%; 25→28 min 45-60%; 28→30 min 60-0%; 30→33 min 0%.

Figure S2 RP-HPLC preparative (A) and analytical (B) analysis of triped FL-2

A

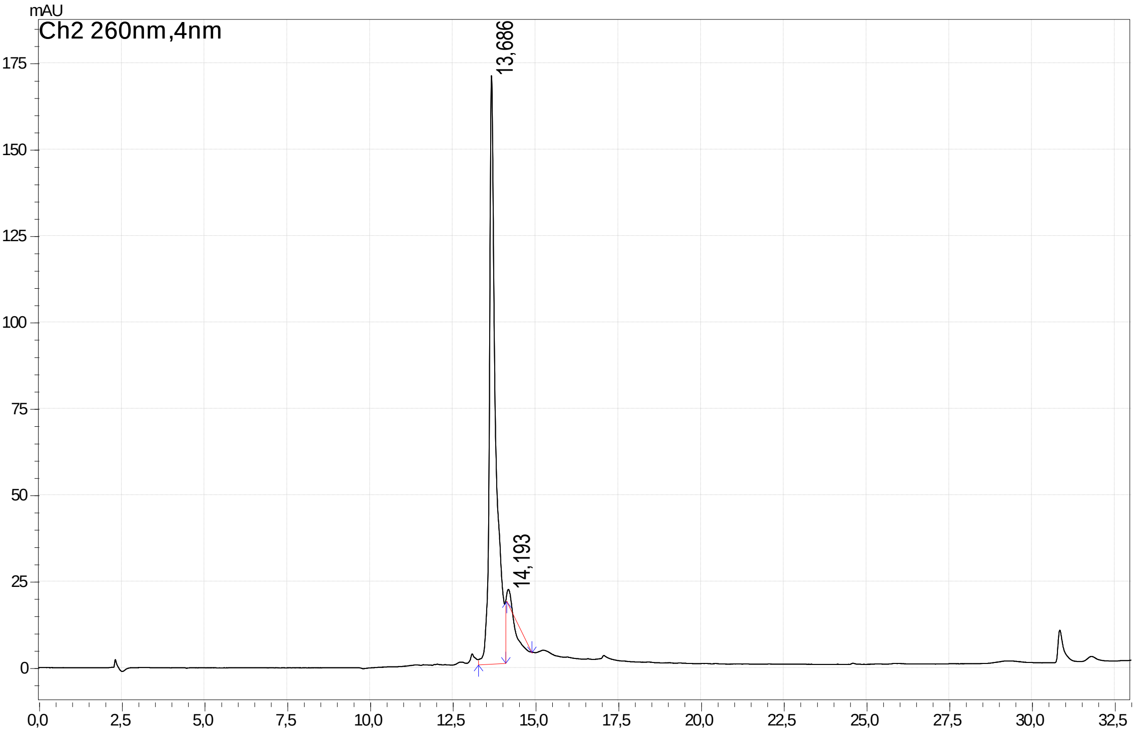

B

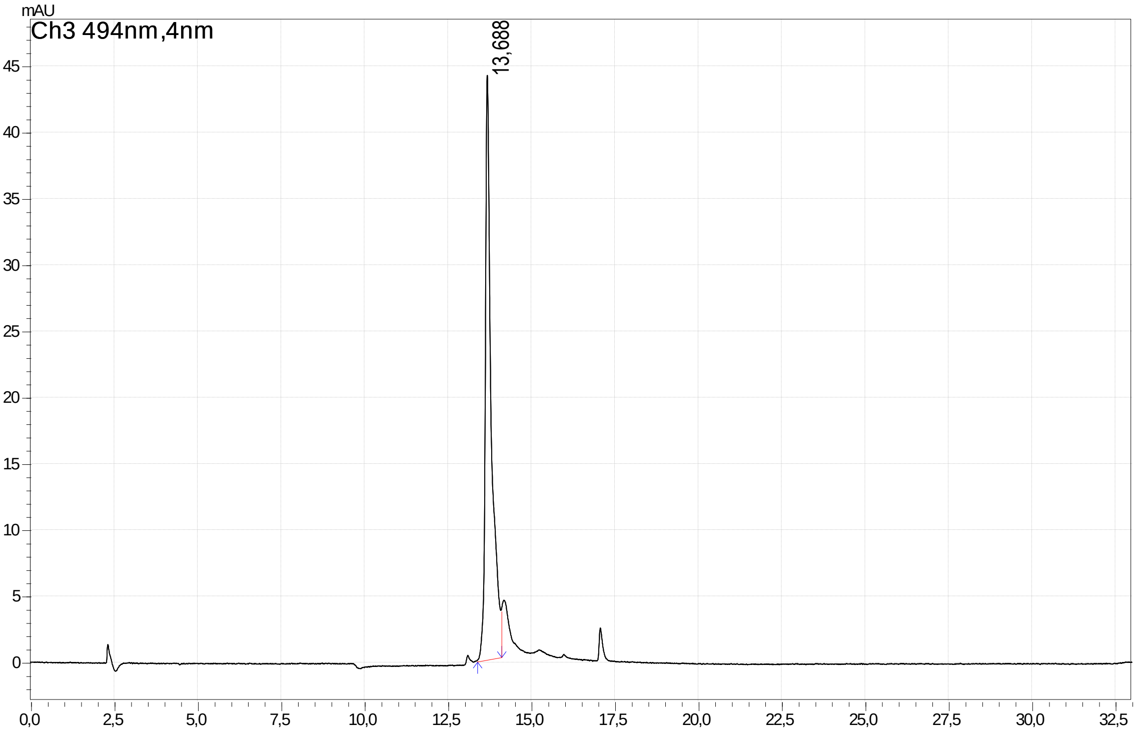

RP-HPLC conditions were as follow: the buffer A (0.1 M CH_3COONH_4) and buffer B (100% CH_3CN). The buffer B gradient: 0→2 min 0%; 2→25 min 0-45%; 25→28 min 45-60%; 28→30 min 60-0%; 30→33 min 0%.

Figure S3 ESI-Q-TOF mass spectrometry analysis of triped **FL-1** (A) and **FL-2** (B).

A) **FL-1.** M.W. calc: 14724.91.; *m/z:* 14718.02

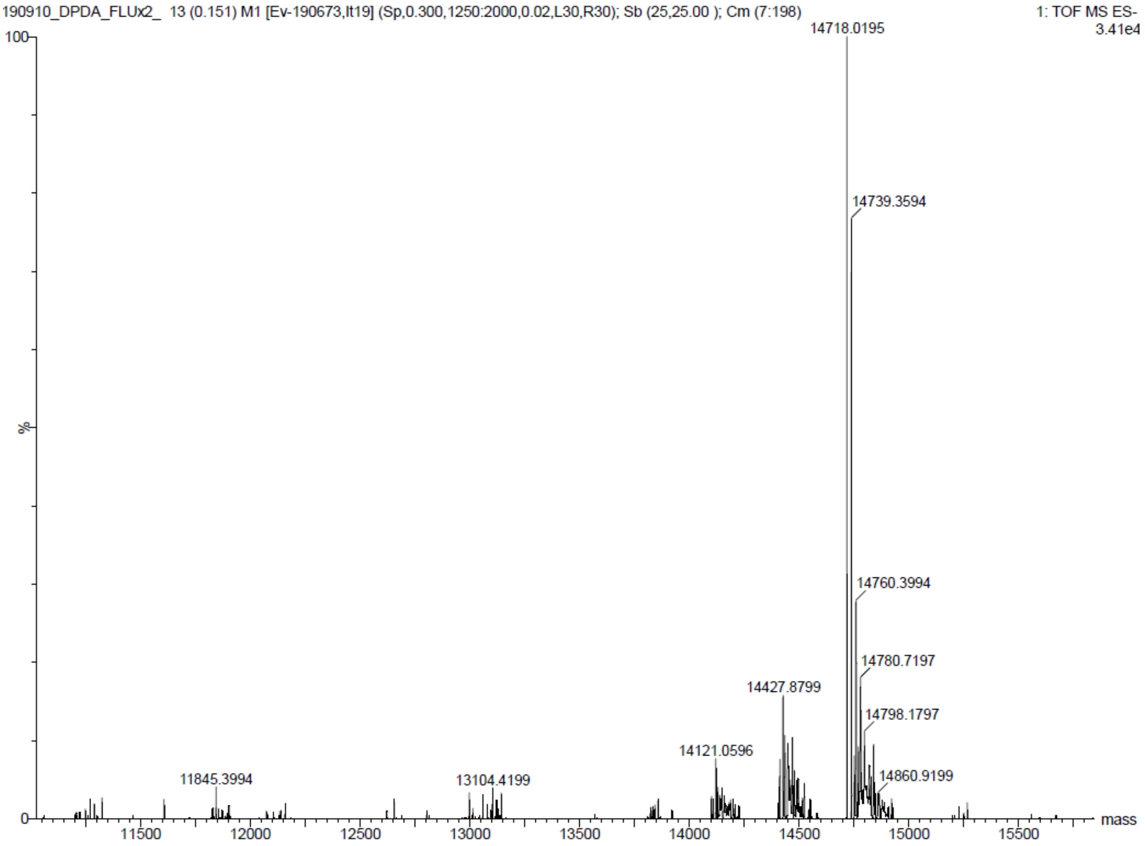

B) **FL-2** M.W. calc: 15215.; *m/z:* 15208.50

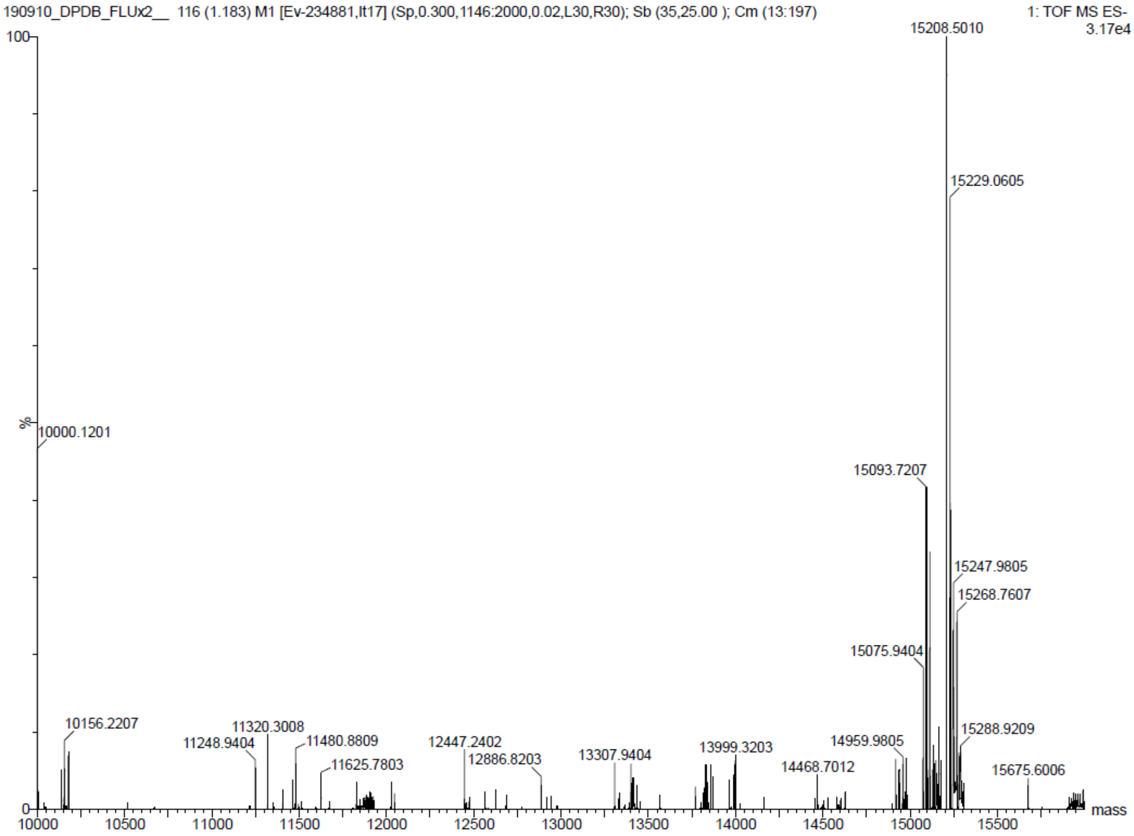

Figure S4 RP-HPLC analytical analysis (A) and ESI-Q-TOF mass spectrometry analysis (B) of ASO-C

A

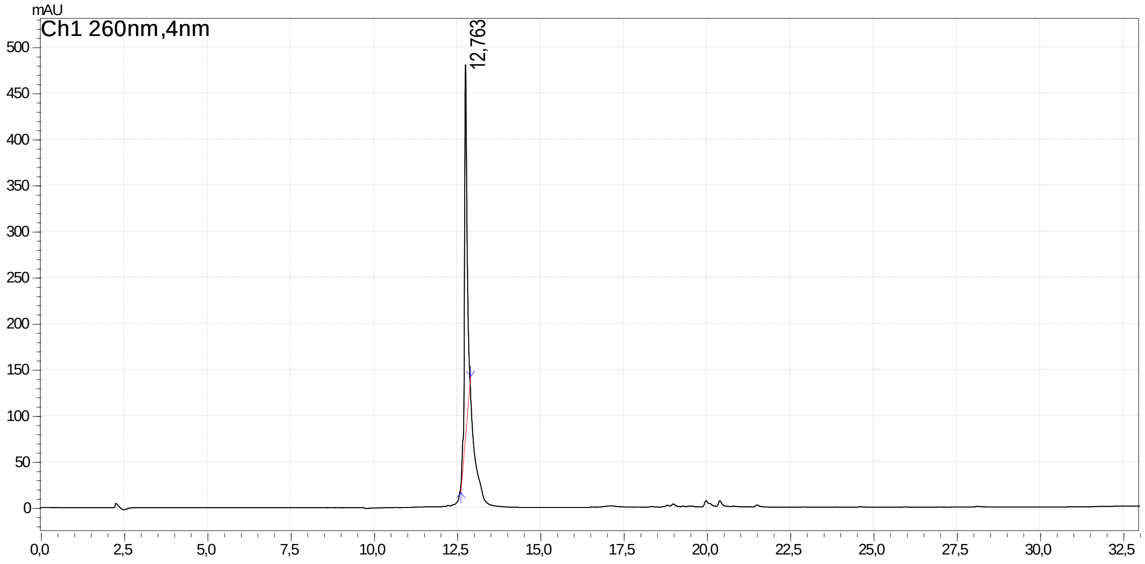

B

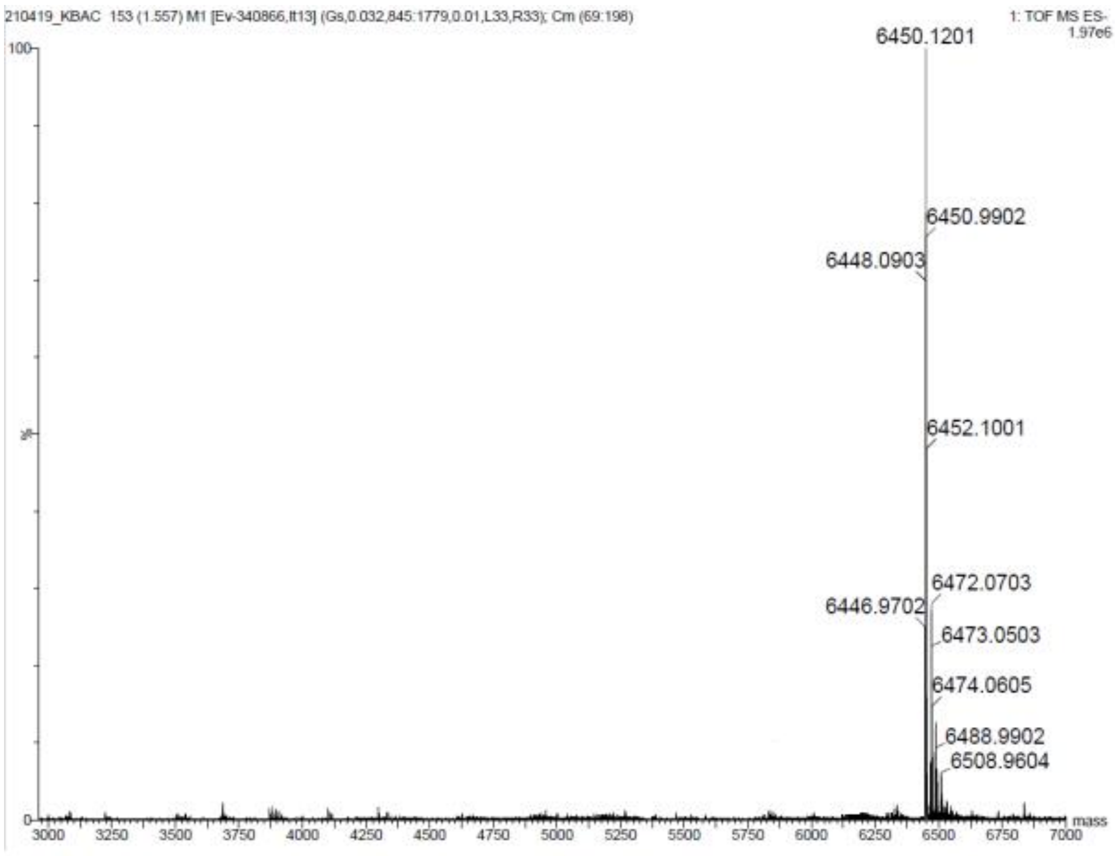

Figure S5. The efficiency of annealing of **1** and **2** (1:1 molar ratio) (lane 2) analyzed by non-denaturating PAGE and visualized by Stains All.

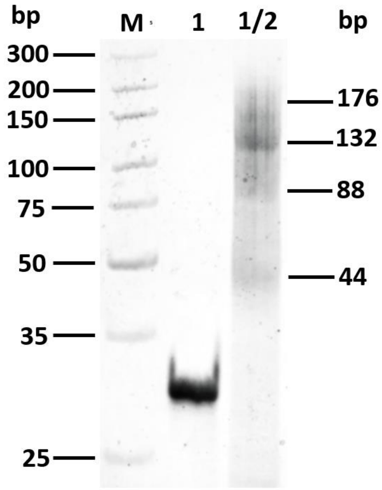

Figure S6. Prolonged incubation time of the cleavage reaction of RNA-1 up to 240 min with RNase H resulted in the increase of the content of 6-nt product for triped **1** and constant level products 9-nt and 7-nt RNA for oligonucleotide AS0-22.

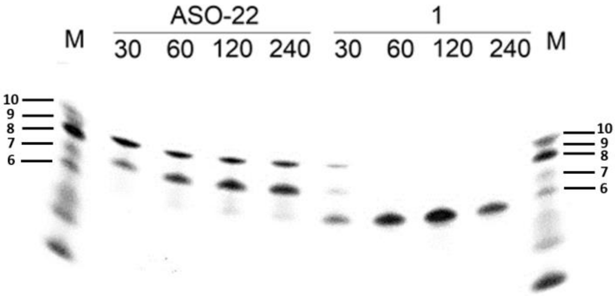

Figure S7. The agarose electrophoretic analysis of the PCR products of the cellular DNA isolated from A431, HeLa, MCF-7 cells amplified with the EZ-PCR ™ Mycoplasma Detection Kit (BI, Cromwell, USA). Commercial negative (**Cn**) and positive controls of mycoplasma (**Cp**) were used for PCR reaction. The lack of the contamination product in DNA of tested cells is demonstrated.

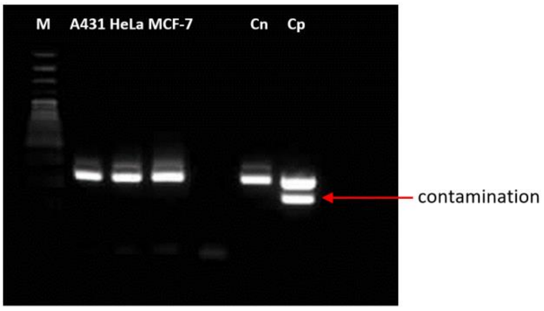

Figure S8. Silencing activity of ASO-22 and **1/2** (50 and 100 nM) towards the exogenous EGFR mRNA monitored by fluorescence microscopy in a dual EGFR-EGFP/RFP fluorescence assay in A431 cells (DFA). The upper row presents the level of a green fluorescence representing the fusion EGFR-EGFP protein and the lower row presents expression of the red fluorescence protein (RFP) (control).

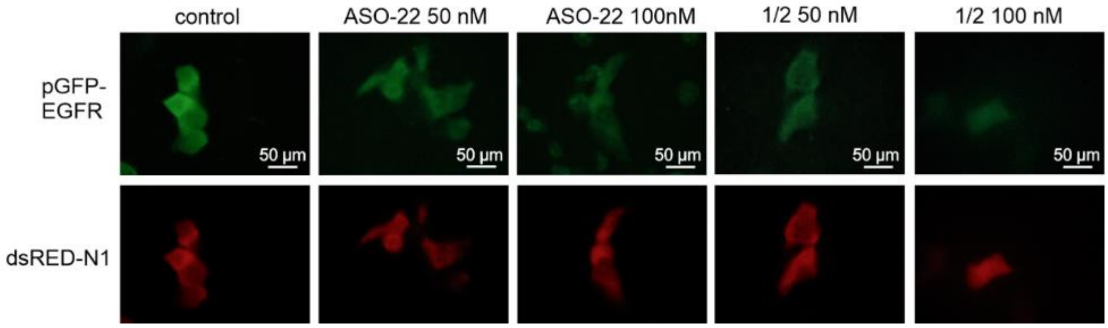

Figure S9. DAPI and FITC filters test demonstrating no emission signals of FITC and DAPI due to the DAPI and FITC fluorophores excitation, respectively. (A) A431 cells transfected with **FL-1/FL-2** (4 µM) cells in the presence of lipofectamine 2000 and analyzed with the FITC filter (exposure time 1s) and checked with the DAPI filter (1s and 2s); (B) A431 cells treated with DAPI (5 µg/mL) and analyzed using a DAPI filter (exposure time 300ms) and checked using a FITC filter (exposure time 300ms and 1s).

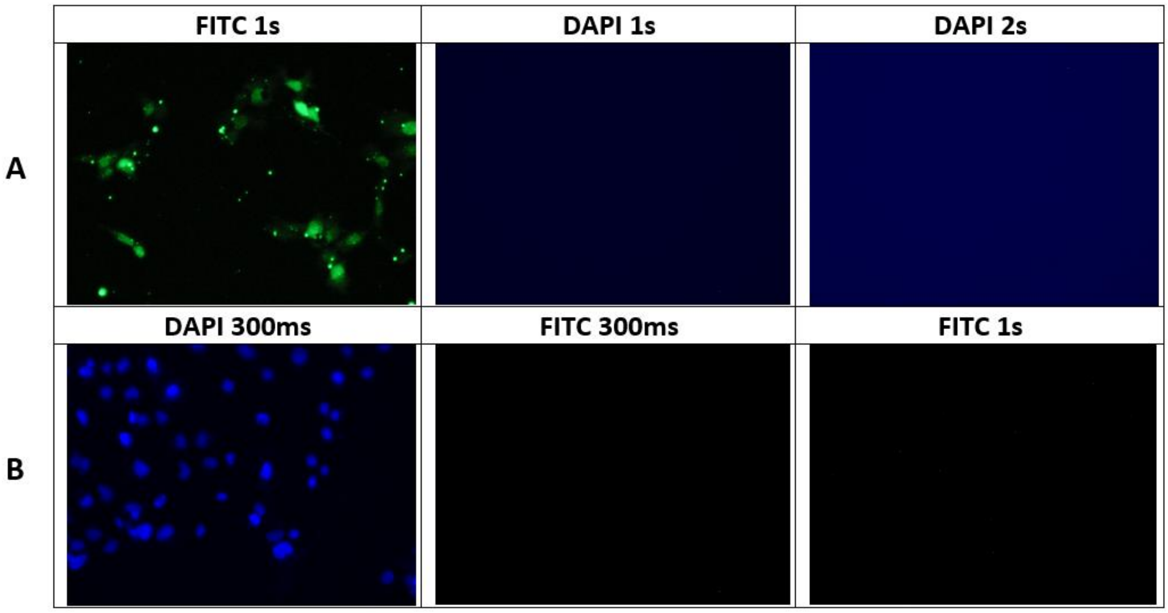

Figure S10. FACS analysis with confidence interval (CI) and the mean with standard deviation (±SD).

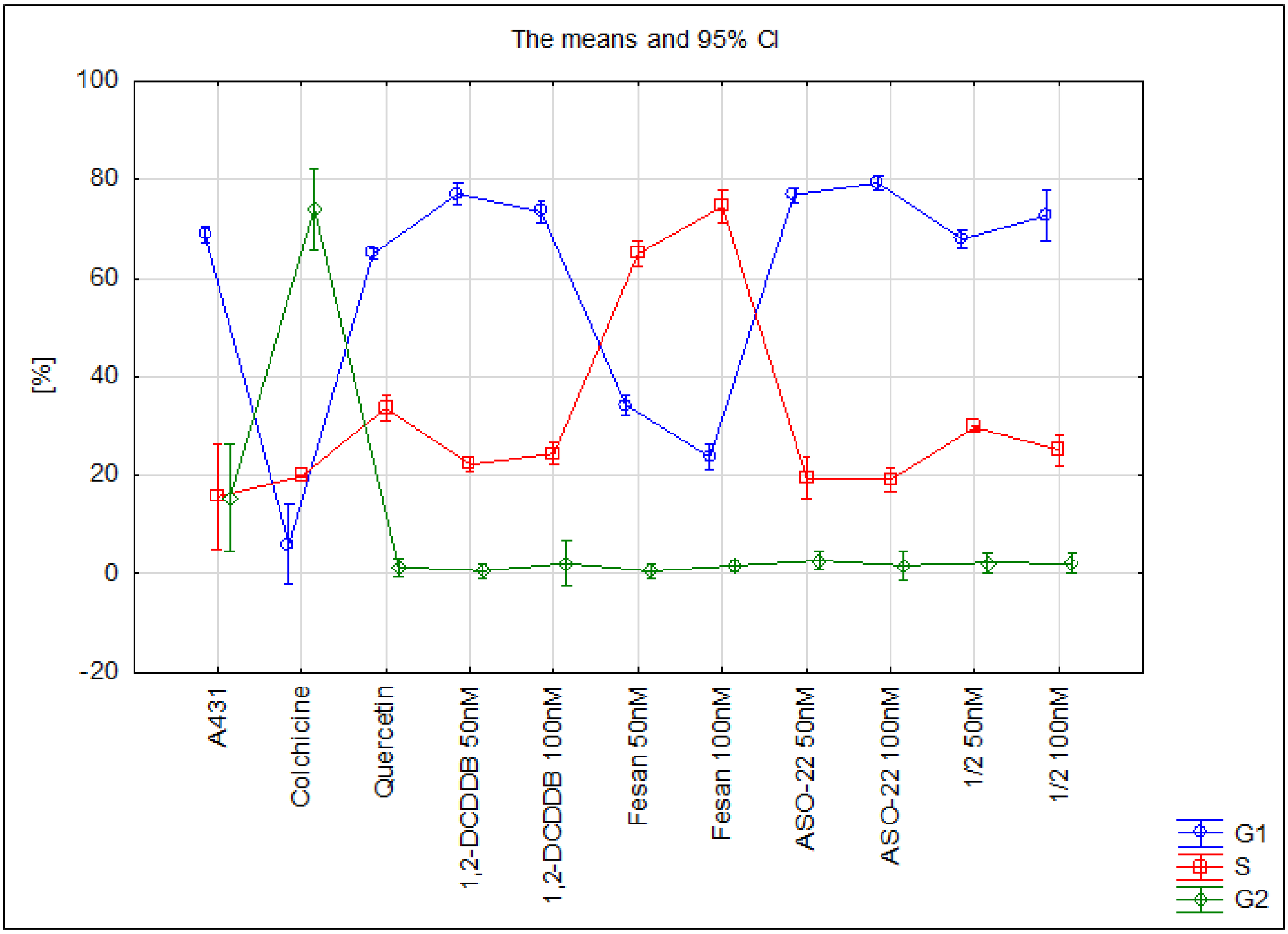

Supplement: Supplementary file 1 [file ijms-22-04863-s001.zip › ijms-1168789-supplementary.pdf]
